# Myelin-oligodendrocyte glycoprotein antibody-positive encephalitis in a patient with Sturge–Weber syndrome

**DOI:** 10.1016/j.radcr.2024.01.006

**Published:** 2024-01-13

**Authors:** Yuko Michishita, Takuya Saito, Tsuyoshi Uchiyama

**Affiliations:** Department of Neurology, Seirei Hamamatsu General Hospital, Hamamatsu, Japan

**Keywords:** Myelin-oligodendrocyte glycoprotein, Sturge-Weber syndrome, Stroke, Magnetic resonance imaging, Autoimmune diseases of the nervous system

## Abstract

Sturge-Weber syndrome (SWS) is a rare congenital disorder associated with systemic vascular malformations characterized by port-wine stains, epilepsy, and glaucoma. Patients with SWS can develop stroke-like symptoms such as hemiparesis. We report a case of a 63-year old woman with SWS who developed left-sided hemiparesis and was finally diagnosed with myelin-oligodendrocyte glycoprotein (MOG) antibody-positive encephalitis. Brain magnetic resonance imaging (MRI) revealed right-dominant bilateral leptomeningeal enhancement, thickened dura mater, and a cerebellar lesion. Cerebrospinal fluid (CSF) examination showed pleocytosis. Both serum and CSF proved positive for MOG antibodies. The patient recovered immediately after intravenous methylprednisolone administration. SWS and MOG antibody-positive encephalitis share similar clinical findings of stroke-like symptoms and leptomeningeal enhancement on MRI. However, MOG antibody-positive encephalitis is highly steroid-responsive in most cases. If a patient with SWS develops stroke-like symptoms accompanied by abnormal CSF findings or subtentorial lesions, testing for MOG antibodies should be considered.

## Introduction

Sturge-Weber syndrome (SWS) is a rare congenital disorder associated with systemic vascular malformations, characterized by port-wine stain, epilepsy, and glaucoma [1]. SWS occurs in 1 in 20,000-50,000 individuals, with equal prevalence in males and females across all races, and is most commonly caused by a somatic mutation in the *GNAQ* gene [2,3]. At least two of the following findings are typically demonstrated among patients with SWS, and these are the key elements for the diagnosis of the disease: port-wine stain, vascular malformations in the brain, and vascular malformations in the eye [4]. Eighty-three percent of patients develop epilepsy as the first presenting manifestation, typically around 6 months of age, and are diagnosed before 1 year of age [5]. Although brain involvement is unilateral in most cases, 14% of patients demonstrate bilateral brain involvement [6]. Patients with SWS may develop stroke-like symptoms, such as hemiparesis, which may persist for several months and are thought to be caused by impaired venous drainage in the affected brain regions. These symptoms may result from seizures or head trauma, or may occur without triggers [7]. However, hemiparesis in a patient with SWS may also be a symptom of another disease. Herein, we report a case of a patient with SWS who developed stroke-like symptoms and was diagnosed with myelin oligodendrocyte glycoprotein antibody-associated disease (MOGAD).

## Case report

**Fig. 1:**
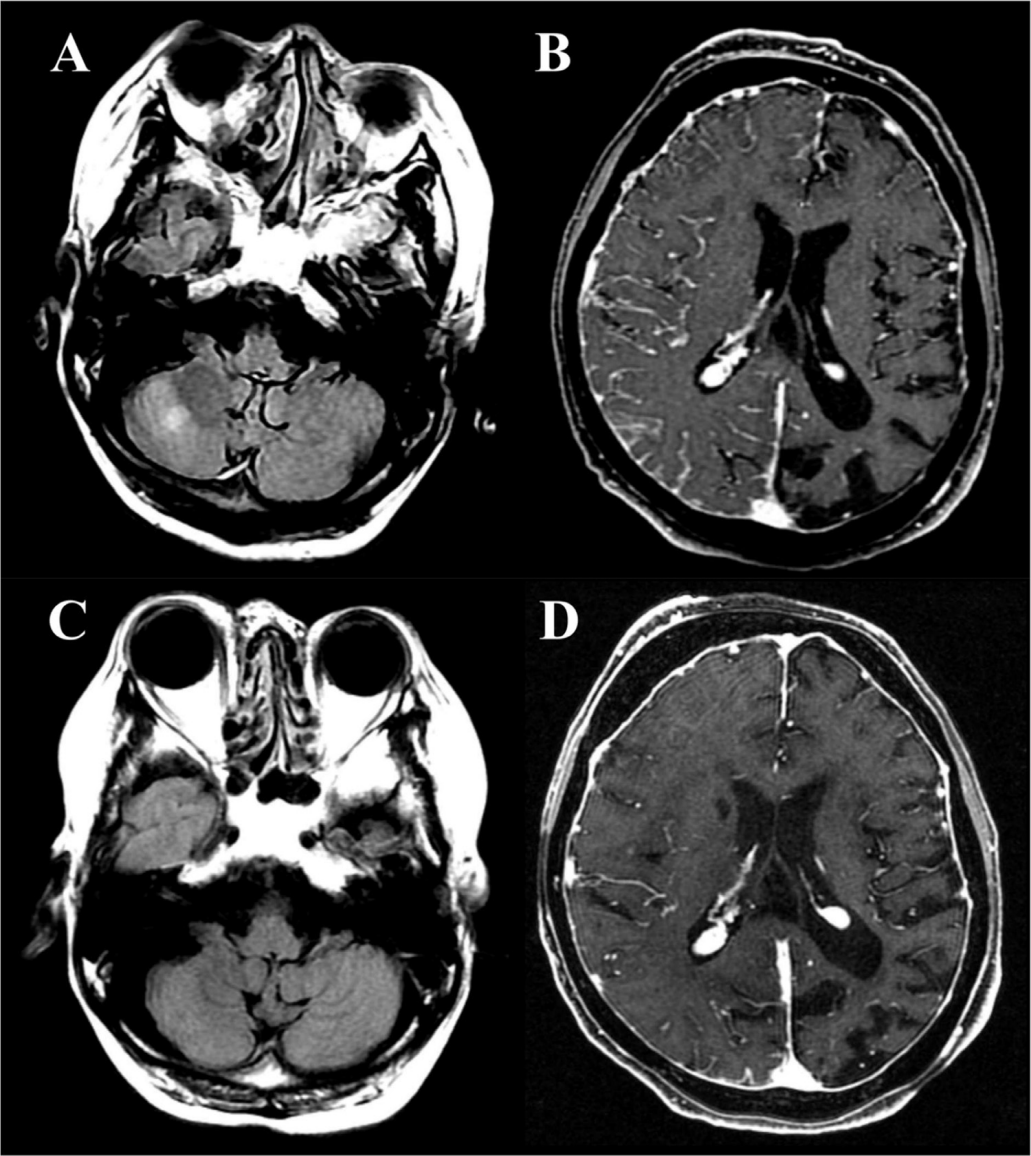
Magnetic resonance imaging findings. Before intravenous methylprednisolone treatment, fluid-attenuated inversion recovery (FLAIR) images revealed a hyperintense lesion in the right cerebellar parenchyma (A). Postcontrast spoiled gradient-recalled echo (SPGR) images revealed right-dominant bilateral leptomeningeal enhancement with thickened dura mater (B). After 5 weeks, the hyperintense lesion in the right cerebellar parenchyma appeared attenuated on FLAIR images (C). While the leptomeningeal enhancement in the right cerebral hemisphere was also attenuated on postcontrast SPGR images, the dura mater thickening persisted (D).

A 63-year old Japanese woman presented with fever, impaired consciousness, conjugated eye deviation to the right, and left-sided hemiparesis. She had a history of SWS, diagnosed by port-wine stains on both sides of her face and extremities, bilateral glaucoma since childhood, and epilepsy, although more detailed information was not available. She was under treatment by her general practitioner but had never attended any neurology department. Cerebrospinal fluid (CSF) examination revealed slight pleocytosis (9 cells/μL; 6 monocytes, 3 granulocytes), elevated protein levels (103 mg/dL), and negative oligo-clonal bands (OCB). Blood sugar, vitamins B1, B12, and folic acid levels, and thyroid and adrenal functions were normal. Screening test results for infectious diseases, including herpes simplex virus, varicella-zoster virus, hepatitis B virus, hepatitis C virus, human immunodeficiency virus, and tuberculosis, were negative. Blood and spinal fluid cultures were negative. Antinuclear antibodies, antineutrophil cytoplasmic antibodies, and antibodies against AQP4, dsDNA, GM1, GQ1b, SS-A, SS-B, sIL-2, GAD, TPO, and thyroglobulin were negative. Both serum and CSF samples were positive for myelin-oligodendrocyte glycoprotein (MOG) antibodies. Brain magnetic resonance imaging (MRI) revealed an abnormal hyperintense lesion in the right cerebellar parenchyma and extensive atrophy of the left hemisphere on fluid-attenuated inversion recovery images. Magnetic resonance angiography revealed hypoplasia of the left middle cerebral artery. Postcontrast spoiled gradient-recalled echo images revealed right-dominant bilateral leptomeningeal enhancements and a thickened dura mater (Fig. 1). Diffusion-weighted imaging revealed no acute ischemic stroke. Cervical spine MRI was normal. Electroencephalography revealed mild diffuse cerebral dysfunction.

Initial treatment with antibacterial and antiviral agents did not improve symptoms. Unconsciousness and left hemiparesis improved rapidly after administration of high-dose intravenous methylprednisolone (IVMP) (1 g for 5 days). Oral prednisolone (20 mg) was administrated after 2 courses of IVMP. The cerebellar lesion and right-sided leptomeningeal enhancement were attenuated after 5 weeks, although the bilateral thickening of the dura mater persisted (Fig. 1). Abnormal CSF findings improved after IVMP.

## Discussion

Herein, we report the case of a patient with SWS who developed hemiparesis. Clinical and radiological findings on admission were similar to the stroke-like symptoms of SWS reported to date. Finally, the patient was diagnosed with MOG antibody-positive encephalitis, and her symptoms improved with steroid administration.

SWS is characterized by leptomeningeal vascular malformations on contrast-enhanced MRI [1]. In addition, brain atrophy in the affected regions occurs over time [1]. In this case, the patient demonstrated port-wine stains, leptomeningeal enhancement on brain MRI, and glaucoma since childhood, which fulfills the 3 key findings for the diagnosis of SWS. Despite the lack of more detailed information about her past medial history, the port-wine stains on both sides and the remaining bilateral thickened dura mater are consistent with the diagnosis of bilateral SWS.

MOGAD is an acute inflammatory demyelinating disorder caused by an antibody that binds to MOG, which is localized in the central nervous system [8]. Leptomeningeal enhancement on contrast-enhanced MRI is a hallmark of cortical encephalitis, a type of MOGAD [9]. A case of MOG antibody-positive unilateral cerebral cortical encephalitis with hemiparesis has been reported [10].

The clinical findings of stroke-like symptoms in patients with SWS and unilateral cerebral cortical encephalitis of MOGAD are similar, and it is difficult to distinguish between them based on clinical findings. SWS and MOGAD show similar leptomeningeal enhancement on brain MRI. However, patients with MOGAD are more likely to have cerebellar and brainstem lesions compared to patients with SWS [11], which may aid in differentiating them from SWS patients. Furthermore, the abnormal CSF findings and a steroid-responsive clinical course suggested autoimmune encephalitis.

Leptomeningeal enhancement is most commonly associated with infectious meningitis, inflammatory diseases such as sarcoidosis, and neoplastic meningitis. Approximately 50% of meningitis exhibit leptomeningeal enhancement, primarily caused by the impairment of the blood-brain barrier. Although MRI findings are sometimes nonspecific, acute bacterial meningitis most commonly exhibits diffuse thin and linear leptomeningeal enhancement [12]. In this case, along with unilateral leptomeningeal enhancement, the negative blood and culture tests for common pathogens decreased the likelihood of bacterial meningitis, leading to the suspicion of SWS involvement and the possibility of MOGAD.

## Conclusion

We report a case of a patient with SWS who developed stroke-like symptoms and was diagnosed with MOG antibody-positive encephalitis. If a patient with SWS presents with stroke-like symptoms, especially those accompanied by subtentorial lesions or abnormal CSF findings, MOGAD complications should be considered.

## Patient consent

A written informed consent was obtained from the patient for the publication of this case report.
